# Pre-service and In-service Teachers’ Metacognitive Knowledge of Learning Strategies

**DOI:** 10.3389/fpsyg.2018.02152

**Published:** 2018-11-09

**Authors:** Vered Halamish

**Affiliations:** School of Education, Bar-Ilan University, Ramat Gan, Israel

**Keywords:** learning strategies, metacognition, teaching, desirable difficulties, metacognitive knowledge

## Abstract

Research in cognitive psychology has suggested that difficulties are often desirable for learning: learning strategies that create difficulties for learners during practice often produce durable learning. Prominent examples of effective learning strategies that introduce desirable difficulties are testing as a means of learning, spacing study sessions over time, and interleaving practice of different topics. Previous research has suggested that, generally, undergraduates’ metacognitive knowledge about the effectiveness of these learning strategies is inaccurate. The goal of the current study was to extend the examination of metacognitive knowledge of learning strategies to pre-service and in-service teachers, and further examine whether teachers’ metacognitive knowledge is related to their teaching experience. Pre-service teachers enrolled in a university teacher training program (*N* = 83) and in-service elementary, junior-high, and high school teachers (*N* = 82) were presented with learning scenarios and predicted which of two learning strategies would yield the better outcome. Results suggested that, overall, both pre-service and in-service teachers failed to predict the advantages of testing, spacing, and interleaving as learning strategies. Furthermore, their knowledge of learning strategies failed to increase with teaching experience. It is, therefore, recommended that explicit instruction about the benefits of empirically supported learning strategies should be included in teacher training and development programs.

## Introduction

In order to learn effectively, learners need to know how to learn effectively. Similarly, to teach effectively, teachers need to know how to do so. It is therefore important to examine whether teachers have accurate metacognitive knowledge of learning strategies.

### Learning Strategies: Desirable Difficulties

Decades of research in cognitive psychology in laboratory settings, and, more recently, in the classroom, has suggested that learning conditions and strategies that speed the rate of acquisition of knowledge may not be conducive to long-term retention and transfer, whereas conditions and strategies that appear to introduce difficulties for learners and slow the rate of acquisition may enhance long-term retention and transfer (Soderstrom and Bjork, 2015). Therefore, difficulties are often desirable for learning (Bjork, 1994; Yan et al., 2017). Overcoming desirabledifficulties appears to trigger cognitive processes that enhance learning (McDaniel and Butler, 2011; Clark and Bjork, 2014). Three of the most effective, robust, and well-studied desirable difficulties, which are clearly relevant to classroom teaching, are using tests as learning events, spacing study sessions over time, and interleaving practice of different topics (Rohrer and Pashler, 2010).

#### Testing as a Means of Learning

Tests are usually used to assess learning, but research suggests that tests can also be used to enhance learning (Rowland, 2014; Adesope et al., 2017; Pan and Rickard, 2018). Taking a test on previously studied information has been shown to produce better long-term learning outcomes than not taking a test or using the same time to restudy the information. This finding is known as the testing effect. A seminal study (Roediger and Karpicke, 2006) compared a group that studied a text over two seven-minute sessions and a second group that studied the text for seven minutes and then spent seven minutes taking a free-recall test. When a final free-recall test was administered after five minutes the former group performed better, but when the test was administered after 2 days or a week the latter group performed better.

#### Spacing Study Sessions

Another highly effective learning strategy is spacing repeated study sessions over time. Although massing (or spacing at shorter intervals) study sessions can enhance short-term performance, distributing study sessions over a longer period with breaks between sessions enhances long-term retention (Carpenter et al., 2012; Kang, 2016). This finding is known as the spacing effect, lag effect, or distributed-practice effect. Carpenter et al. (2009), for example, demonstrated the benefits of spacing in educational settings. Eighth graders received a review session either 1 or 16 weeks after a course on U.S. history. A test conducted 36 weeks after the review session revealed better test performance when the review was conducted 16 weeks after the topic was initially studied.

#### Interleaving Practice of Different Topics

A related effective strategy involves interleaving the practice of various topics, rather than blocking practice by topic. Research suggests that blocking produces more rapid and errorless learning, whereas interleaving produces better learning outcomes in the long run (Kang, 2017). Benefits of interleaving have been demonstrated for diverse learning tasks, such as procedural learning, verbal learning, math learning and inductive learning (Rohrer, 2012). In a study on inductive learning, for example, Kornell and Bjork (2008) presented participants with paintings by different artists along with the painter’s name in blocks—all paintings by a given painter presented consecutively—or interleaved. Later on, participants were presented with new paintings by the same painters and were asked to identify the painter. The results revealed better inductive learning with interleaved study than with blocked study.

### Metacognitive Understanding of Learning Strategies

Recent studies have suggested that undergraduates’ metacognitive knowledge about the effectiveness of learning strategies such as testing, spacing, and interleaving, is often inaccurate (Bjork et al., 2013). McCabe (2011) presented undergraduates with concrete learning scenarios and asked them to predict which of two strategies would yield better educational outcomes for each scenario. The scenarios were based on previously published studies and the two strategies used in each scenario included one empirically shown to be effective and one empirically shown to be less effective. Two of the scenarios required participants to choose between testing and restudying and between interleaving and blocking. Participants’ predictions were inaccurate: only 30 and 7% of the participants accurately predicted the relative benefits of testing and interleaving, respectively. Using a similar procedure, Morehead et al. (2016) found that 49 and 16% of undergraduates accurately predicted the relative benefits of testing and interleaving, respectively. They also developed a learning scenario involving spacing and found that 69% of undergraduates accurately predicted the relative benefits of spacing over massing. Morehead et al. (2016) also surveyed university instructors and revealed that they were somewhat more accurate than undergraduates, but the difference was small. Specifically, 62, 74, and 13% of the university instructors accurately predicted the relative benefits of testing, spacing, and interleaving, respectively.

### The Current Study

The goal of the current study was to extend the examination of the metacognitive knowledge of learning strategies to pre-service and in-service teachers, and further examine whether teachers’ knowledge is related to their teaching experience. Although university students tend not to have accurate metacognitive knowledge of learning strategies, it might be that in-service teachers have acquired such knowledge through their teaching experience.

## Materials and Methods

### Participants

Participants were 83 pre-service teachers and 82 in-service teachers.

The *pre-service teachers* were enrolled in a university teacher training program. They completed the survey anonymously and voluntarily online as a class demonstration at the very beginning of the first lesson of a course. Participants were informed that the data might also be used for research purposes and were asked to indicate whether they consented to their responses being used for that purpose. One additional participant did not consent and was excluded from all analyses.

The *in-service teachers* completed the survey anonymously and voluntarily online following an invitation that was posted in online teacher discussion groups or distributed by several teachers among their colleagues. They were informed that the data is collected for research purposes by the author and consent was implied by taking the survey.

Participants were asked to indicate whether they were indeed in-service teachers. Two additional respondents were excluded because they indicated that they were not. According to their self-report, the in-service teachers were of a diverse range of subjects at elementary school (26%), junior-high school (33%), high school (39%) or other schools (2%). Their mean length of tenure was 13.91 years (range 0–43).

### Procedure

A three-scenario survey was administered in which participants were asked to predict which of two learning strategies would yield better outcomes (see Supplementary Materials). The testing and interleaving scenarios were drawn from previous surveys (McCabe, 2011^1^; Morehead et al., 2016). The spacing scenario was developed for the current study. Each scenario was based on a previously published, well-cited, educationally relevant study (testing scenario: the 1-week condition from Roediger and Karpicke, 2006 Experiment 1; spacing scenario: the test/study condition from Carpenter et al., 2009; interleaving scenario: Kornell and Bjork, 2008, Experiment 1b) and described two learning strategies, one empirically validated and one not, as well as a description of a criterion test.

In each scenario, participants used a seven-point scale (1-7) to indicate how much more effective they thought one strategy would be relative to the other, as measured by subsequent test scores. In the spacing and interleaving scenarios, a score of seven represented a strong belief that the empirically validated strategy would be more effective, whereas in the testing scenario the scale was the other way round.

This study was carried out in accordance with the recommendations of the institutional review board, the departmental ethics committee, and the ethical principles of the American Psychological Association. Approval by an ethics committee was not required according to the institutional and departmental guidelines. Participants’ consent was obtained as described above in the Participants section.

## Results

The testing scenario scores were reversed, so that in all scenarios higher ratings (≥5) indicated a belief that the empirically validated strategy would be more effective and lower ratings (≤3) indicated a belief that the other strategy would be more effective. A combined score of an overall belief in the superior effectiveness of the validated strategies was computed by averaging the ratings for the three scenarios. Table 1 presents the descriptive and inferential statistics by scenario and group. Supplementary Table 1 presents the distribution of responses by group and scenario.

**Table 1:** Descriptive and inferential statistics by group and by scenario.

	*Pre-service teachers*			*In-service teachers*			*Pre-service versus in-service teachers*
	
Scenario	% Correct	*M* (SD)	Mean score vs. the neutral score (4)	% Correct	*M* (SD)	Mean score vs. the neutral score (4)	
Testing vs. restudying	49	3.88 (2.48)	*t*(82) = 0.44, *p* = 0.659, Cohen’s *d* = 0.05	48	3.85 (2.34)	*t*(81) = 0.57, *p* = 0.573, Cohen’s *d* = 0.06	*t*(163) = 0.07, *p* = 0.945, Cohen’s *d* = 0.01
Longer vs. shorter spacing	28	3.12 (2.06)	*t*(82) = 3.89, *p* = 0.000, Cohen’s *d* = 0.43	40	3.94 (2.10)	*t*(81) = 0.26, *p* = 0.794, Cohen’s *d* = 0.03	*t*(163) = 2.52, *p* = 0.013, Cohen’s *d* = 0.40
Interleaving vs. blocking	23	2.71 (2.27)	*t*(82) = 5.18, *p* = 0.000, Cohen’s *d* = 0.57	12	2.30 (1.73)	*t*(81) = 8.89, *p* = 0.000, Cohen’s *d* = 0.98	*t*(163) = 1.29, *p* = 0.197, Cohen’s *d* = 0.20
Combined score		3.24 (1.22)	*t*(82) = 5.71, *p* < 0.001, Cohen’s *d* = 1.26		3.37 (1.27)	*t*(81) = 4.52, *p* < 0.001, Cohen’s *d* = 1.01	*t*(163) = 0.67, *p* = 0.507, Cohen’s *d* = 0.10

*Scores greater than 4 indicate a belief in the relative effectiveness of the empirically supported strategy. % Correct is the percentage of participants who chose a score of “5” or higher.*

To examine whether participants understood the benefits of effective learning strategies, ratings were compared with the neutral response (4, i.e., a prediction that the two strategies would result in similar test scores). Pre-service teachers’ mean rating was not significantly different from the neutral response in the case of testing, and was significantly lower in the cases of spacing, interleaving, and the combined score. In-service teachers’ mean rating was not significantly different than the neutral response in the cases of testing and spacing and was significantly lower than the neutral response in the case of interleaving and the combined score.

Next, we examined the relationship between teaching experience and metacognitive knowledge. First, the ratings of the pre-service and in-service teachers were compared. In the case of spacing, the ratings were significantly lower for pre-service teachers than for in-service teachers. This is not to say that in-service teachers understood the benefits of spacing; rather that the pre-service teachers erroneously predicted the opposite effect. There were no significant differences in the ratings of the two groups in the case of testing, interleaving, and the combined score. Second, the relationship between in-service teachers’ length of tenure and metacognitive knowledge of learning strategies was analyzed. The results are presented in Table 2. The Spearman’s rank order correlation between the combined score and the in-service teachers’ length of tenure was negative and significant, suggesting that more experienced teachers were *less* likely to predict the relative effectiveness of learning strategies correctly. Correlations between length of tenure and ratings for individual scenarios were negative in absolute terms, but not significant except in the case of the spacing scenario.

**Table 2:** Spearman rank-order correlations between scenario ratings and length of tenure for in-service teachers.

Scenario	Length of tenure (years)
(1) Testing vs. restudying	*r_s_* = −0.16, *p* = 0.159
(2) Longer vs. shorter spacing	*r_s_* = −0.31, *p* = 0.006
(3) Interleaving vs. blocking	*r_s_* = −0.20, *p* = 0.082
(4) Combined score	*r_s_* = −36, *p* = 0.001

*N = 79.*

## Discussion

The current study focused on pre-service and in-service teachers’ metacognitive knowledge of three well-established learning strategies that present desirable difficulties—testing, spacing, and interleaving. The results suggest that both pre-service and in-service teachers failed to predict the benefits of testing, spacing, and interleaving. In fact, pre-service teachers predicted that shorter spacing would be more effective than longer spacing, and both pre-service and in-service teachers predicted that blocking would be more effective than interleaving.

It is informative to compare the teachers’ metacognitive knowledge to that of university instructors, as reported by Morehead et al. (2016). Both pre-service and in-service teachers in the current study failed to predict the benefits of testing and spacing (correct predictions: 49 and 28% respectively for pre-service teachers; 48 and 40% for in-service teachers), whereas the majority of university instructors in the study of Morehead et al. (2016) did predict the benefits of these strategies (correct predictions: 62 and 74%, respectively). In the case of interleaving the two studies produced similar results: the benefits of interleaving were predicted by 23% of pre-service teachers and 12% of in-service teachers in this study and 13% of university instructors in Morehead et al. (2016) study. Overall, these results suggest that k-12 pre-service and in-service teachers have less metacognitive knowledge of effective learning strategies than university instructors.

These results are worrisome. If teachers do not understand the benefits of effective learning strategies they cannot be expected to apply them in their teaching, let alone teach students to use them in independent study. These results might explain, for example, why undergraduates do not have a good metacognitive knowledge of effective learning strategies (McCabe, 2011): their teachers probably did not expose them to or teach them about the effectiveness of such strategies, simply because they themselves did not recognize it. This is a shame because McCabe (2011) demonstrated that it is possible to teach students to appreciate the benefits of effective strategies by giving them explicit information about such strategies. Future research might examine the effectiveness of a similar intervention with teachers.

The results also suggest that metacognitive knowledge of learning strategies does not improve with teaching experience. Overall, in-service teachers were no better than pre-service teachers at predicting the benefits of testing, spacing, and interleaving. Furthermore, in-service teachers’ metacognitive knowledge of desirable difficulties was negatively related to teaching experience. In other words, more experienced teachers were actually less likely to predict the benefits of desirable difficulties. The sources of this negative relationship are yet to be explored in a future study, perhaps with a more representative sample of teachers.

One limitation of the current study is that it focused on teachers’ metacognitive beliefs but not on their actual teaching decisions in the classroom. Another avenue for future research is to examine the extent to which teachers use effective learning strategies that are desirable difficulties in the classroom, and the relationship between their metacognitive knowledge and the strategies they actually use for teaching.

## Conclusion and Recommendations

Research has long suggested that some of the most well-recognized effective learning strategies are counterintuitive, since they improve long-term educational outcomes despite posing difficulties during the learning process (Bjork, 1994; Clark and Bjork, 2014). The results of this study suggest that pre-service and in-service teachers often have a poor understanding of the effectiveness of these strategies. It is, therefore, recommended that explicit instruction about the benefits of empirically supported learning strategies should be included in teacher training programs and continuing professional development programs for in-service teachers in order to encourage teachers to incorporate them into their teaching and promote them to their students.

## Ethics Statement

This study was carried out in accordance with the recommendations of the ethics committee of Bar-Ilan University and the ethical principles of the American Psychological Association (APA). An ethics approval was not required as per Bar-Ilan University’s guidelines and national guidelines. Since the study involved only an anonymous questionnaire and was assumed not to create distress or harm, informed consent was not required according to the APA ethical principles. Nevertheless, participants’ consent was obtained as follows: (1) the pre-service teachers completed the survey anonymously and voluntarily online in class as a class demonstration; they were informed that the data might also be used for research purposes and were asked to explicitly indicate within the survey whether they consented to their responses being used for that purpose, and (2) the in-service teachers completed the survey remotely, anonymously and voluntarily online; They were informed that the data is collected for research purposes by the author and their consent was obtained by virtue of survey completion.

## Author Contributions

VH conceived, designed and conducted the study, analyzed the data, and wrote the manuscript.

## Conflict of Interest Statement

The author declares that the research was conducted in the absence of any commercial or financial relationships that could be construed as a potential conflict of interest.

## Footnotes

1 I thank Jennifer McCabe for sharing her materials with me.
