# Supplementary material for: Pre-service and In-service Teachers’ Metacognitive Knowledge of Learning Strategies
Source: Front Psychol. 2018 Nov 9;9:2152. doi: 10.3389/fpsyg.2018.02152 (PMC6238295; doi:10.3389/fpsyg.2018.02152)
Supplement: Supplementary file 1 [file Data_Sheet_1.docx]

***Supplementary Materials***

# Pre-Service and In-Service Teachers' Metacognitive Knowledge of Learning Strategies

**Learning scenarios used in the survey (translated from Hebrew)**

**1. Testing versus restudying**

In two different classes, students learned a 275-word text. In one class, students first read the text for 7 minutes, and then were asked to write down from memory as much of the material from the text as they could for another 7 minutes (“test” class). In the other class, students first read the text for 7 minutes, and then they read it again for another 7 minutes (“rereading” class). After one week, students from both classes were asked to write down as much of the text as they could remember. Which class do you think remembered the text better?

| 1 | 2 | 3 | 4 | 5 | 6 | 7 |
| --- | --- | --- | --- | --- | --- | --- |
| “test” class remembered much better |  |  |  |  |  | “rereading” class remembered much better |

**2. Longer versus shorter spacing**

In two different classes, students spent several lessons studying a specific history topic. Later on, both classes had a review lesson in which they answered a series of questions about the topic and then received a sheet giving the correct answers. One class had the review lesson a week after they had finished studying the topic (“week later” class) and the other class had the review lesson 16 weeks after they had finished studying the topic (“16 weeks later” class). Thirty-six weeks after their review lesson both classes were tested on the topic. The test included the questions from the review lesson as well as new questions. Which class do you think got higher grades on the test?

| 1 | 2 | 3 | 4 | 5 | 6 | 7 |
| --- | --- | --- | --- | --- | --- | --- |
| “week later” class got much higher grades |  |  |  |  |  | “16 weeks later” class got much higher grades |

**3. Interleaving versus blocking**

An art teacher presented his students with paintings by 12 painters. He presented six different paintings by each painter (for a total of 72 paintings). In one class, the teacher presented all six paintings by one painter, then all six paintings by another painter, and so on (“consecutive” class). In the other class, the teacher presented the paintings of the various painters mixed together, so that one painting by one artist was followed by another painting by a different artist, and so on (“mixed” class). After presenting all the paintings the teacher presented his students with new paintings by the same 12 painters and asked them to identify the painter in each case. Which class do you think did better at identifying the painters?

| 1 | 2 | 3 | 4 | 5 | 6 | 7 |
| --- | --- | --- | --- | --- | --- | --- |
| “consecutive” class did much better |  |  |  |  |  | “mixed" class did much better |
