# Supplementary material for: Pre-service and In-service Teachers’ Metacognitive Knowledge of Learning Strategies
Source: Front Psychol. 2018 Nov 9;9:2152. doi: 10.3389/fpsyg.2018.02152 (PMC6238295; doi:10.3389/fpsyg.2018.02152)
Supplement: Supplementary file 2 [file Data_Sheet_2.docx]

Supplementary Table 1

*Distribution of responses (in percentages) by group and scenario*

|  |  | *Pre-service teachers* | | | | | | |  | *In-service teachers* | | | | | | |
| --- | --- | --- | --- | --- | --- | --- | --- | --- | --- | --- | --- | --- | --- | --- | --- | --- |
| Scenario |  | 1 | 2 | 3 | 4 | 5 | 6 | 7 |  | 1 | 2 | 3 | 4 | 5 | 6 | 7 |
| Testing vs. restudying |  | 28.92 | 15.66 | 6.02 | 0 | 8.43 | 19.28 | 21.69 |  | 26.83 | 14.63 | 4.88 | 6.10 | 12.20 | 18.29 | 17.07 |
| Longer vs. shorter spacing |  | 32.53 | 15.66 | 14.46 | 9.64 | 8.43 | 10.84 | 8.43 |  | 18.29 | 14.63 | 7.32 | 19.51 | 10.98 | 13.41 | 15.85 |
| Interleaving vs. blocking |  | 45.78 | 20.48 | 10.84 | 0 | 2.41 | 3.61 | 16.87 |  | 42.68 | 30.49 | 7.32 | 7.32 | 3.66 | 2.44 | 6.10 |

For each scenario, a score of 7 represents a strong belief that the empirically validated strategy would be more effective whereas a score of 1 represents a strong belief that the other strategy would be more effective.
